# A Translational Roadmap for Neurological Nonsense Mutation Disorders

**DOI:** 10.3390/ijms27031418

**Published:** 2026-01-30

**Authors:** Jiaqing Li, Zhenyun Zhu, Sanqing Xu

**Affiliations:** Department of Pediatrics, Tongji Hospital, Tongji Medical College, Huazhong University of Science and Technology, Wuhan 430030, China; li.jiaqing@tjh.tjmu.edu.cn (J.L.); yunerduoduo@tjh.tjmu.edu.cn (Z.Z.)

**Keywords:** stop codon readthrough, nonsense mutation, precision medicine, readthrough therapy, translational framework

## Abstract

Nonsense mutations, responsible for ~11% of gene lesions causing human monogenic diseases, introduce premature termination codons (PTCs) that lead to truncated proteins and nonsense-mediated mRNA decay (NMD). In the central nervous system (CNS), these mutations drive severe, progressive neurological conditions such as spinal muscular atrophy, Rett syndrome, and Duchenne muscular dystrophy. Readthrough therapies—strategies to override PTCs and restore full-length protein expression—have evolved from early aminoglycosides to modern precision tools including suppressor tRNAs, RNA editing, and CRISPR-based platforms. Yet clinical translation remains hampered by inefficient CNS delivery, variable efficacy, and the absence of personalized stratification. In this review, we propose a translational framework—the 4 Ds of Readthrough Therapy—to systematically address these barriers. The framework dissects the pipeline into Detection (precision patient identification and biomarker profiling), Delivery (engineered vectors for CNS targeting), Decoding (context-aware molecular correction), and Durability (long-term safety and efficacy). By integrating advances in machine learning, nanocarriers, base editing, and adaptive trial designs, this roadmap provides a structured strategy to bridge the translational gap. We advocate that a synergistic, modality-tailored approach will transform nonsense suppression from palliative care to durable, precision-based cures for once-untreatable neurological disorders.

## 1. Introduction: The Promise and Peril of Nonsense Suppression

Nonsense mutations, which generate premature termination codons (PTCs) within protein-coding sequences, are a catastrophic cause of inherited neurological disorders [1,2]. By truncating essential proteins and triggering nonsense-mediated mRNA decay (NMD), these mutations disrupt synaptic integrity, neuronal survival, and myelination, leading to progressive and often irreversible neurological decline. Nonsense mutations affect a significant proportion of genetic disorders with major neurological or neuromuscular manifestations, including spinal muscular atrophy (SMA), Rett syndrome, Duchenne muscular dystrophy (DMD), and genetic epileptic encephalopathies, thus the clinical need for effective interventions is urgent and unmet [3,4,5].

To address this, readthrough therapy has emerged as a promising precision medicine approach. It utilizes molecular agents to “suppress” the ribosome into bypassing these premature stop signals, allowing the cell to continue translation and produce a full-length, functional protein. The concept of translational readthrough—forcing the ribosome to bypass a PTC and synthesize a full-length protein—has captivated researchers for decades [6,7]. The therapeutic landscape has expanded from serendipitously discovered aminoglycosides to rationally designed small molecules [8,9], engineered suppressor tRNAs (sup-tRNAs), RNA editing platforms, and CRISPR-based gene correction [10,11,12]. Yet, the journey from bench to bedside has been fraught with setbacks. High-profile clinical failures, such as the mixed outcomes of ataluren in DMD, underscore a critical translational gap [13]. Variable efficacy, poor blood–brain barrier (BBB) penetration, off-target effects, and a one-size-fits-all approach have stymied progress [14].

**Figure 1:**
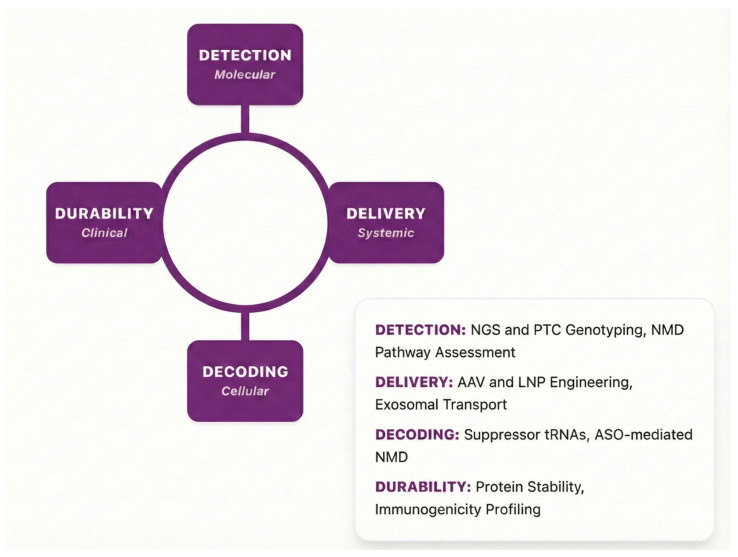
The 4 Ds translational framework for nonsense mutation therapy: A schematic overview of the integrated “4 Ds” framework for developing readthrough therapies for neurological nonsense mutation disorders. The framework comprises four interconnected pillars: Detection (molecular-level patient identification and stratification), Delivery (systemic engineering of vehicles for CNS targeting), Decoding (cellular-level precision correction of premature termination codons), and Durability (clinical-level assurance of long-term safety and efficacy). Specific components within each pillar are listed: Detection includes next-generation sequencing (NGS), PTC genotyping, and nonsense-mediated decay (NMD) pathway assessment; Delivery encompasses AAV and lipid nanoparticle (LNP) engineering and exosomal transport systems; Decoding involves suppressor tRNAs and antisense oligonucleotide (ASO)-mediated NMD inhibition; Durability focuses on protein stability and immunogenicity profiling.

To overcome this stagnation, we propose a paradigm shift from a modality-centric view to a translational pipeline perspective. This review introduces and elaborates the “4 Ds of Readthrough Therapy” framework: Detection, Delivery, Decoding, and Durability. Unlike general precision medicine frameworks that focus on broad diagnostic–therapeutic pairings, the “4 Ds” model is primarily an operational and predictive framework specifically designed for the unique molecular hurdles of nonsense suppression. It advances beyond existing translational models by explicitly linking the molecular detection of codon context and NMD efficiency with the selection of a decoding modality and the necessity for brain-penetrant delivery systems. By doing so, this model serves as a structured roadmap to diagnose specific translational bottlenecks, integrate emerging technologies, and accelerate the development of personalized, effective treatments for nonsense mutation disorders of the nervous system (Figure 1).

## 2. Detection: Precision Patient Identification and Stratification

The first critical step in the translational pipeline is accurately identifying patients who will benefit from readthrough and predicting their therapeutic response. This moves beyond simple genotyping to a multi-parametric profiling approach.

### 2.1. Beyond the Genotype: Codon Context and Transcriptomics

Readthrough efficiency is profoundly influenced by the nucleotide sequence surrounding the PTC (the “codon context”), the identity of the stop codon (UGA, UAG, UAA), and downstream exon junction complexes that govern NMD susceptibility [15]. Among disease-causing nonsense mutations, the relative frequencies are approximately 38.5% for UGA, 40.4% for UAG, and 21.1% for UAA [16]. Generally, UGA is the “leakiest” and most susceptible to pharmacological readthrough, followed by UAG and UAA [17,18,19,20]. Machine learning models trained on ribosome profiling and deep mutational scanning data can now predict mutation-specific readthrough potential, enabling pre-therapeutic stratification [21]. Recent studies demonstrate that NMD efficiency varies significantly among patients, influencing both baseline transcript levels and therapeutic response [22].

### 2.2. Biomarker Development and Patient-Derived Models

The lack of robust, quantifiable biomarkers for target engagement and functional protein restoration has plagued clinical trials [23]. Next-generation mass spectrometry now allows for the direct detection of low-level, full-length proteins in patient cerebrospinal fluid or via extracellular vesicles (EVs) [24]. These lipid bilayer nanoparticles, secreted by all CNS cell types, can traverse the BBB into the peripheral circulation while carrying a molecular cargo (proteins, nucleic acids) that mirrors the physiological state of their parent cells. In the context of nonsense suppression, the mass spectrometry-based detection of full-length proteins within CNS-derived EVs in blood or plasma provides a “liquid biopsy” to monitor therapeutic efficacy and protein restoration without the need for invasive brain biopsies. Meanwhile, patient-derived induced pluripotent stem cells (iPSCs) differentiated into cortical neurons, motor neurons, or 3D brain organoids provide a revolutionary platform [25,26]. These “patients-in-a-dish” models enable the high-throughput screening of readthrough compounds in a genetically authentic, human neural environment, de-risking drug candidates before clinical entry [27].

### 2.3. Integrating Multi-Omic Data for a Therapeutic Decision Matrix

The future of Detection lies in integrative diagnostics: combining genomic data (mutation, context), transcriptomic data (NMD efficiency, tRNA expression profiles), and proteomic readouts from iPSC models. This integrated profile can guide the selection of the optimal therapeutic modality—small molecule, sup-tRNA, or gene editing—for each individual, marking the true dawn of precision readthrough.

## 3. Delivery: Engineering Vehicles to Conquer the Blood–Brain Barrier

One of the most formidable obstacles to treating central nervous system (CNS) disorders with readthrough therapies is the restrictive nature of the BBB. The BBB is composed of tightly joined endothelial cells, astrocytic end-feet, and pericytes that collectively prevent the entry of most macromolecules and therapeutic agents into the brain parenchyma. This severely limits the efficacy of systemically administered small molecules, ASOs, or RNA-based therapies for neurological conditions [28]. Overcoming this requires innovative bioengineering.

### 3.1. Evolution of CNS-Capable Nanocarriers

Lipid nanoparticles (LNPs), pivotal for mRNA vaccine delivery, are being repurposed for the brain. By functionalizing LNPs with BBB-penetrating peptides (e.g., targeting transferrin or LDL receptors), researchers can achieve receptor-mediated transcytosis [29]. Polymeric nanoparticles, such as those made from PLGA, offer sustained release and can be similarly targeted [30]. Engineered exosomes [31], inherently cross the BBB and can be loaded with RNAs (ASOs, guide RNAs, sup-tRNAs) and decorated with neuron-specific targeting motifs, dramatically enhancing brain accumulation while minimizing immunogenicity [32]. Recent advances in focused ultrasound with microbubbles provide a physical method to transiently open the BBB for therapeutic delivery [33].

### 3.2. Viral Vectors and Route of Administration

Adeno-associated virus (AAV) vectors remain a mainstay for one-time gene therapy, but their immunogenicity, cargo limits, and preferential tropism are limitations [34]. Next-generation capsid engineering [35], using directed evolution in primate models, is yielding novel AAV variants with enhanced CNS tropism and reduced seroprevalence [35]. To bypass the restrictive BBB, these engineered capsids are designed to utilize receptor-mediated transcytosis, allowing them to cross from the vasculature into the brain parenchyma. While early variants like AAV-PHP.eB showed high CNS tropism in specific mouse strains by targeting the LY6A receptor—a mechanism not conserved in primates—recent efforts have identified primate-tropic variants such as AAV.CAP-B10 that exhibit superior distribution in the non-human primate brain. Furthermore, new capsids are being engineered to target human-specific receptors like Carbonic Anhydrase IV, which is highly expressed in brain endothelial cells, ensuring more reliable clinical translation. Alternatively, intranasal administration [36] offers a direct, non-invasive route to the brain via the olfactory and trigeminal nerves, bypassing the BBB entirely for oligonucleotides and small molecules [36]. Engineered virus-like particles (VLPs) represent another promising platform for efficient protein delivery without viral genome integration [35].

### 3.3. The Delivery–Decoding Interface

Effective Delivery is not generic; it must be tailored to the Decoding modality. This dictates that the vector’s persistence, tissue tropism, immunogenicity, and cargo capacity align with the therapeutic agent’s mechanism of action, required duration of activity, and molecular size. For example, sup-tRNAs require persistent expression to maintain therapeutic levels of the rescued protein, favoring AAV or integrating RNA vectors [37,38]. Conversely, CRISPR-based editors are designed as precise, transient catalysts where prolonged expression heightens the risk of off-target effects; for these agents, biodegradable delivery systems like LNPs, which facilitate a controlled, finite burst of activity, represent the optimal strategy [35]. This fundamental principle extends to other modalities, governing the development of next-generation ASO conjugates engineered to enhance central nervous system residency and bridge pharmacokinetic gaps [39,40]. Thus, a successful therapeutic strategy demands that the delivery platform is not merely a passive carrier but is co-optimized with the decoding modality, ensuring that the molecular precision engineered at the bench effectively translates to target engagement and functional correction at the bedside.

## 4. Decoding: Achieving Molecular Precision at the Ribosome

This pillar confronts the core therapeutic challenge: how to force the ribosome to bypass PTC with both efficiency and fidelity. The evolution from broad-spectrum compounds to context-aware, high-specificity molecular machines marks a critical paradigm shift.

### 4.1. The Renaissance of Small Molecule Design

Pharmacological agents that induce translational readthrough represent a promising, therapeutic approach for diseases caused by nonsense mutations. These compounds, such as aminoglycosides (e.g., Gentamicin, G418), next-generation synthetic aminoglycoside derivatives (e.g., ELX-02), and non-aminoglycoside small molecules like Ataluren (PTC124), promote the insertion of near-cognate tRNAs at PTCs, thereby allowing ribosomes to bypass the stop signal and produce full-length, potentially functional proteins.

Aminoglycosides were the first class of compounds identified to induce ribosomal readthrough by promoting the misincorporation of near-cognate tRNAs [10,41]. Since the landmark discovery of aminoglycosides G418 and paromomycin as readthrough compounds in 1985, other aminoglycosides, including gentamicin, neomycin, and paromomycin, have been tested for their ability to suppress PTCs. Their readthrough efficacies vary widely (1% to 25%), depending on the chemical composition, stop codon identity, and surrounding nucleotide context [6,19,20]. Moreover, the long-term use of aminoglycosides is limited by side effects including dose-dependent nephrotoxicity, ototoxicity [42,43], and the induction of translational errors [44]. Newer synthetic aminoglycosides have been developed for enhanced selectivity and reduced toxicity. One such compound, ELX-02, is a eukaryote-optimized aminoglycoside derivative that demonstrates improved ribosome specificity. Preclinical studies have shown that ELX-02 can restore CFTR activity in patient-derived organoids and stabilize *CFTR* mRNA by modulating NMD [45,46]. However, results from a Phase II trial (NCT04135495) in CF patients showed variable outcomes, likely due to heterogeneity in stop codon context, individual pharmacodynamics, and the incomplete suppression of the NMD pathway.

Considerable effort has been devoted to identifying non-aminoglycoside compounds with readthrough-inducing activity. The most well known is ataluren (PTC124), an oxadiazole compound that enhances readthrough by interfering with the interaction between release factors and the ribosome [11]. Ataluren received conditional approval from the European Medicines Agency (EMA) for treating nonsense mutation-associated DMD, based on data showing modest benefits in walking ability and slowed disease progression (NCT00592553). However, subsequent Phase III trials (NCT03179631, NCT01826487) failed to meet primary efficacy endpoints, raising questions about patient stratification, dose optimization, and context-dependent responsiveness [13,47]. Beyond ataluren, several novel compounds are under investigation. For instance, 2,6-diaminopurine (DAP) enhances readthrough by inhibiting FTSJ1, a tRNA-specific methyltransferase, thereby altering tRNA modification and decoding fidelity [48] and cytosine analogs and compounds like clitocine have shown the ability to increase PTC suppression by modifying tRNA structure or enhancing its interaction with the ribosome [49]. RTC13, RTC14, and NV848 are newer small molecules identified through high-throughput screening that exhibit readthrough activity in preclinical models with reduced toxicity compared with aminoglycosides [50,51].

To date, over 30 pharmacological compounds with PTC readthrough activity have been characterized through in vitro and in vivo models. The overall clinical translation remains limited, primarily due to low and context-dependent readthrough efficacy, nonspecific amino acid incorporation at the PTC site, and off-target effects impacting global translation [7,14]. The new generation focuses on rational design informed by cryo-EM ribosomal structures. Compounds are being engineered to stabilize specific ribosome-near-cognate tRNA interactions at PTCs while minimizing interference at natural termination codons (NTCs) [21,48]. Furthermore, the concurrent administration of a readthrough inducer with a modulator of the NMD pathway—such as SMG1 inhibitors or an ASO that blocks the recruitment of UPF1—can create a synergistic effect, dramatically increasing the steady-state pool of target mRNA and thereby amplifying the yield of full-length proteins [52,53]. For many complex, multi-domain neuronal proteins, the translation of a full-length mRNA is insufficient; the nascent polypeptide must fold correctly, traverse cellular compartments, and achieve stable conformation. This introduces a third therapeutic layer: pharmacological chaperones. These compounds bind to and stabilize the correctly folded state of the rescued protein or assist in its trafficking, ensuring that translational rescue translates to durable functional restoration at the synapse or organelle level [54].

### 4.2. The Rise in Nucleic Acid Therapies

**Figure 2:**
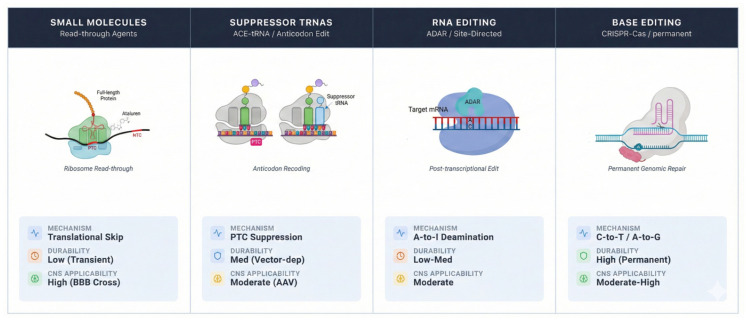
Therapeutic modalities for premature termination codon (PTC) readthrough: Overview of the four principal therapeutic strategies for nonsense mutation suppression. (1) Small Molecule Therapies: Pharmacological agents (e.g., Ataluren, aminoglycosides, ELX-02) that promote ribosomal readthrough by facilitating the incorporation of a near-cognate tRNA at the PTC, enabling synthesis of full-length proteins. (2) Suppressor tRNAs (sup-tRNAs): Engineered tRNA genes delivered via viral or non-viral vectors that are aminoacylated and decode the PTC as a sense codon, restoring translation of the full-length native protein. (3) RNA Editing: Endogenous adenosine deaminases acting on RNA (ADAR) recruited by engineered guide RNAs (e.g., circular RNAs in the LEAPER system) to catalyze the deamination of adenosine to inosine (read as guanosine) within a stop codon, thereby converting it into a sense codon at the transcript level. (4) DNA Editing Technologies: CRISPR-based precision genome editing tools, such as adenine base editors (ABEs), which directly convert an A-T to a G-C base pair at the genomic DNA level to correct the PTC (e.g., TAG→TGG) without inducing double-strand breaks, offering a permanent, one-time corrective solution. The schematic highlights the molecular mechanism, site of action (ribosomal, transcriptomic, or genomic), and key components of each therapeutic modality within the nonsense suppression toolkit.

The field has witnessed the ascent of nucleic acid-based platforms, which offer a fundamentally distinct strategy by directly programming the cellular machinery to correct or bypass the error at the RNA or DNA level, often with nucleotide-level precision (Figure 2). Among these, sup-tRNAs represent a targeted genetic supplementation approach [37,55,56]. This strategy involves delivering an engineered tRNA gene designed to decode a specific PTC as a sense codon [12]. These tRNAs have demonstrated promising results across a range of genetic disorders, including DMD [57], CF [37,38,58], and CDKL5 deficiency disorders [59]. Sup-tRNAs have also been shown to antagonize NMD, increasing therapeutic effect by stabilizing PTC-containing mRNA [58]. Modern high-throughput functional profiling platforms, such as tRNA-ScreenSeq, enable the screening of thousands of tRNA variants to identify those with optimal suppression efficiency and, critically, minimal off-target recognition of NTCs [37,38]. The latest innovations are focused on enhancing this specificity through precise anticodon loop engineering and the incorporation of chemical modifications that improve molecular stability and reduce immunogenicity [60].

A complementary and reversible strategy is RNA editing, which corrects the PTC at the transcript level without altering the genome [61,62]. The most clinically advanced systems harness endogenous Adenosine Deaminases Acting on RNA (ADAR), which mediate the enzymatic conversion of adenosine (A) to inosine (I)—read as guanine (G) by the ribosome. This process can potentially correct nonsense mutations by converting stop codons (e.g., UGA) into sense codons (e.g., UGG for tryptophan), thereby allowing full-length protein production [63,64,65]. Emerging RNA-editing platforms such as REPAIR [61] and RESCUE [66], use guide RNAs to recruit ADAR enzymes to specific RNA sites. These systems have successfully corrected nonsense mutations in models of Rett syndrome [67], and DMD [62]. The appeal of this approach lies in its transient, RNA-targeted nature, which avoids permanent genomic changes. However, significant challenges remain in achieving a uniformly high editing efficiency in vivo across diverse tissues and in minimizing promiscuous bystander editing at non-target adenosines.

For a permanent, one-time curative intervention, CRISPR-based DNA editing systems offer the potential for direct genomic correction. Promising results have been observed in a wide range of disease models, including DMD [68,69,70,71,72,73], CF [74,75], Leber congenital amaurosis [76], Alzheimer’s disease [77], Huntington’s disease [78], and amyotrophic lateral sclerosis [79]. Moving beyond classic Cas9-mediated excision and error-prone repair pathways, base editing has emerged as a particularly promising tool for nonsense mutations [75,80]. Adenine Base Editors (ABEs) can directly convert an A-T base pair to a G-C pair, enabling the precise correction of PTCs like TAG to TGG or CAG without introducing double-strand DNA breaks or requiring donor DNA templates, thereby significantly improving the safety and efficiency profile of genomic correction [75,80]. For mutations that are not amenable to direct base editing, CRISPR-mediated transcriptional modulation provides a powerful alternative. This can involve using catalytically dead Cas9 (dCas9) fused to transcriptional activators to upregulate the expression of a healthy homologous allele or a functionally related gene, or employing CRISPR interference (CRISPRi) to knockdown key NMD factors, thereby stabilizing the mutant transcript and increasing its availability for other readthrough mechanisms [52].

### 4.3. A Modality Selection Framework

The Decoding pillar provides a diverse therapeutic toolkit, but its clinical success hinges on selecting the optimal modality for each specific patient and mutation context. This selection process must evolve from empirical trial-and-error to a rational, data-driven decision matrix informed by the comprehensive Detection profile. A rigorous framework should integrate multiple layers of patient-specific data to match the inherent advantages and limitations of each decoding strategy to the unique biological and clinical scenario. At the genomic level, the precise identity of the stop codon (UGA, UAG, UAA) and its immediate nucleotide context are primary determinants. These sequence features directly influence the predicted efficacy of small molecules, the design of suppressor tRNAs, and the feasibility of RNA or base editing approaches [15,21,48].

Beyond sequencing, the cellular and systemic context dictates feasibility. The genomic accessibility and chromatin state of the target locus critically impact the efficiency of CRISPR-mediated DNA editing. For RNA-level interventions, the endogenous expression levels of essential components—such as the abundance of specific ADAR isoforms for RNA editing or the cellular tRNA pool that a suppressor tRNA must compete with—must be considered [38,60]. The spatial requirement for protein restoration is equally vital: does the disease pathophysiology require global protein expression across diverse cell types in the CNS, or is correction within a specific neuronal subtype sufficient? This question guides the delivery strategy but also the modality choice; for example, a broadly expressed sup-tRNA delivered via a pan-neuronal AAV serotype might be suitable for global disorders, whereas a locally administered, self-replicating RNA vector encoding a base editor could be engineered for focal correction. By systematically evaluating these parameters, clinicians and researchers can move toward a future of “prescription readthrough,” where the decoding modality is not a one-size-fits-all solution but a precisely matched therapeutic component within the larger 4D framework.

## 5. Durability: Ensuring Long-Term Efficacy and Safety

The ultimate success of any readthrough therapy is measured not by transient biomarker changes but by sustained functional benefit and an acceptable long-term safety profile. Achieving Durability requires navigating a complex landscape where the initial molecular correction must be maintained over decades in non-dividing neuronal populations without eliciting detrimental immune responses, cumulative toxicity, or loss of the therapeutic effect. This pillar addresses the chronic challenges that determine whether a promising intervention translates into a viable cure.

### 5.1. Navigating Immune Recognition and Response

The administration of exogenous genetic material [28,39] or engineered macromolecules invariably poses an immunogenic risk that can undermine durability through the neutralization of the therapeutic agent or the induction of an inflammatory pathology. Viral vectors can trigger both pre-existing and de novo humoral and cell-mediated immune responses against the viral capsid, potentially limiting re-administration and, in some cases, causing the cytotoxic T-lymphocyte-mediated clearance of transduced cells [34,81]. Similarly, bacterial-derived proteins such as Cas9 from *Streptococcus pyogenes* contain epitopes that can be recognized by human memory T-cells, posing risks of inflammatory reactions and reduced editing efficiency upon repeated exposure [82]. Strategies to mitigate these risks are multi-faceted. For editing enzymes, the humanization of protein sequences or the use of smaller, orthologous Cas variants (e.g., Cas12f, CasMINI) derived from non-pathogenic bacteria can minimize recognition by the adaptive immune system [83,84]. Furthermore, transient pharmacological immunosuppression regimens co-administered with the therapy, or the innovative concept of inducing antigen-specific immune tolerance through tolerogenic vaccination prior to treatment, are under investigation to create a permissive immunological window for durable engraftment [82].

### 5.2. Achieving and Maintaining Specificity

The risk of off-target effects poses a persistent threat to durability, especially in post-mitotic neurons where errors are permanent and cumulative [85]. For CRISPR-mediated genome editing, the deployment of high-fidelity Cas9 variants and comprehensive off-target screening methods like CIRCLE-seq has become standard practice to minimize genomic collateral damage [85]. The field of RNA editing faces its own specificity challenge, where the promiscuous activity of adenosine deaminase enzymes could lead to widespread transcriptome alterations [86]. Enhancing specificity requires engineering the enzyme-guide RNA complex. Systems like CLUSTER (Cas13 Leveraged for Undesired Sequence Toggling and Editing Reduction) employ dual-guide requirements to activate editing, thereby drastically increasing specificity by demanding two adjacent target sites for catalytic activity [87]. Additionally, the development of deactivated, “dead” ADAR2 variants that must be fused to a highly specific RNA-binding domain to regain activity offers another path to confine edits exclusively to the intended transcript [88].

### 5.3. The Inextricable Link Between Delivery and Longevity

The durability of a therapeutic effect is fundamentally governed by its delivery mechanism, creating a nexus that must be carefully evaluated. The AAV-mediated delivery of gene editing components or suppressor tRNA genes aims for a one-time, durable correction by achieving stable transduction [8,89]. However, this permanence carries the inherent risk of irreversible off-target edits or genotoxic events [90]. Conversely, the non-viral delivery of transient agents, such as lipid nanoparticle-encapsulated mRNA encoding an editor or the repeated administration of ASOs, offers a titratable and potentially reversible approach. This paradigm allows for dosing adjustments and halting therapy if adverse events occur but necessitates chronic treatment regimens with associated compliance and access challenges [29,90]. The choice between these strategies is not generic but must be informed by a risk–benefit calculus that considers the disease’s natural history, the patient’s age, and the specific window for therapeutic intervention to achieve lasting neurological benefits.

## 6. Integrating the 4 Ds: A Translational Roadmap to Clinical Implementation

**Figure 3:**
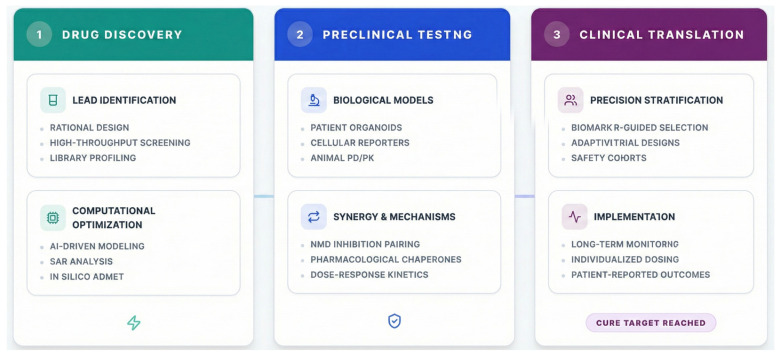
Pipeline for readthrough therapy development and clinical translation: Roadmap outlining the translational pipeline from discovery to clinical implementation of readthrough therapies. The pipeline is divided into three main phases: (1) Drug Discovery, involving rational design, high-throughput screening, and computational/AI-driven optimization of lead compounds. (2) Preclinical Testing, utilizing advanced biological models including patient-derived organoids, cellular models, and animal studies. This phase emphasizes combination therapies and mechanistic synergy, such as pairing NMD inhibitors with pharmacological chaperones. (3) Clinical Trials, featuring biomarker-guided patient stratification, adaptive trial designs, and protocols for long-term monitoring and individualized therapy. The pipeline emphasizes an iterative, data-driven approach integrating molecular insights with clinical outcomes.

The ultimate value of the 4 Ds framework lies not in its conceptual separation but in its systematic integration (Figure 3). Synthesizing these pillars provides an actionable, end-to-end roadmap to navigate the complex journey from mechanistic discovery to transformative clinical application.

### 6.1. Preclinical Validation Through the Integrated Lens

The preclinical development of any novel readthrough therapeutic technique must be re-envisioned through the integrated 4D model from its inception. This begins with rigorous Detection-phase validation in functionally relevant, patient-derived models, such as iPSC-derived neurons or cerebral organoids harboring the specific nonsense mutation, ensuring the intervention corrects the molecular defect in its native human cellular context [25,26]. Concurrently, Delivery must be rigorously assessed not in isolation but within models that recapitulate the pertinent biological barrier. This involves utilizing advanced systems such as microfluidic blood–brain barrier-on-a-chip platforms or relevant transgenic animal models to quantitatively evaluate central nervous system biodistribution, target tissue penetration, and functional engagement [29,36]. The Decoding efficacy—measured as the restoration of full-length, functional proteins—must be evaluated alongside a comprehensive off-target profile using transcriptomic and proteomic analyses to ensure specificity [14]. Finally, Durability assessments require long-term studies in humanized animal models to monitor the persistence of the therapeutic effect, the potential for immune clearance, and the long-term safety of any genetic or epigenetic alterations [89,90]. This multi-faceted preclinical package de-risks clinical translation by ensuring no critical pillar is overlooked.

### 6.2. Designing Next-Generation, Framework-Informed Clinical Trials

The failure of traditional, one-size-fits-all clinical trial designs in rare, genetically heterogeneous neurodevelopmental disorders necessitates a paradigm shift informed by the 4 Ds principle. Future trials must embrace biomarker-enriched adaptive designs, where Detection-derived biomarkers—such as quantifiable target protein in cerebrospinal fluid or digital readouts of neuronal function—serve as intermediate endpoints [23]. Current patient stratification relies heavily on genomic sequencing to identify the specific PTC and its surrounding 3′ context [15,21]. Emerging biomarkers for clinical trials include the measurement of NMD efficiency via transcriptomic analysis and the quantification of residual protein levels in patient-derived iPSCs or CNS-derived EVs [22]. These biomarkers allow for the real-time, data-driven adaptation of dosing, patient stratification, or even therapeutic modality during the trial itself. Furthermore, the framework supports the logic of mutation-focused basket trials or N-of-1 studies, where a specific therapeutic agent (e.g., a suppressor tRNA engineered for a particular UGA context) is tested across a small cohort of patients sharing that precise genetic lesion, regardless of their clinical diagnosis [38,75]. This maximizes the signal-to-noise ratio and accelerates the path to approval for ultra-personalized interventions. Importantly, the framework provides a rational basis for combination therapy trials from the outset. Rather than testing single agents in sequence, trials can be designed to evaluate synergistic regimens—such as a readthrough inducer paired with an NMD modulator and a pharmacological chaperone—where preclinical data from integrated models suggest complementary mechanisms of action to enhance Durability and efficacy [8,54].

### 6.3. Navigating Regulatory Pathways and Ensuring Equitable Access

The personalized and technologically complex nature of next-generation readthrough therapies presents novel challenges for regulatory science and global health equity. Regulatory agencies will require new frameworks to evaluate integrated therapeutic packages where the drug product may be a unique combination of a delivery vector and a patient-specific nucleic acid payload. The development of standardized Detection biomarker toolkits and validated Delivery efficiency metrics will be crucial as surrogate endpoints to demonstrate biological activity, especially for disorders with slow clinical progression [23]. From an access perspective, the field must proactively address the daunting specter of cost and manufacturing complexity. Strategies such as platform manufacturing for modular delivery systems, the development of in vivo editing techniques that avoid ex vivo cell manipulation, and international pre-competitive consortia for data and resource sharing will be essential to translate these scientific breakthroughs into accessible cures for patients worldwide, ensuring the 4 Ds roadmap leads not just to regulatory approval but to real-world impact [39,91].

## 7. Conclusions: From Framework to Future Cures

The 4 Ds framework provides a holistic, structured approach to overcoming translational barriers in nonsense mutation disorders. By integrating precision Detection, tailored Delivery, context-aware Decoding, and Durability-focused design, the field can shift from palliative care to curative, personalized therapies. The convergence of advanced biomolecular tools, AI-driven stratification, and adaptive clinical designs will transform the promise of readthrough into life-altering reality for patients with neurological genetic disorders.

The next decade will see a shift from broad, poorly effective compounds to a new generation of “prescription readthrough”—where a patient’s integrated Detection profile leads to a prescribed, customized combination of Delivery and Decoding technologies, monitored for Durability. By embracing this integrated roadmap, the field can transform the enduring promise of nonsense suppression into a tangible, life-altering reality for patients with neurological genetic disorders.

## Data Availability

No new data were created or analyzed in this study. Data sharing is not applicable to this article.

## Abbreviations

The following abbreviations are used in this manuscript:

ASO	Antisense oligonucleotide
ADAR	Adenosine deaminases acting on the RNA
AAV	Adeno-associated virus
ABEs	Adenine base editors
BBB	Blood–brain barrier
CNS	Central nervous system
DMD	Duchenne muscular dystrophy
EMA	European Medicines Agency
EVs	Extracellular vesicles
iPSC	Induced pluripotent stem cell
LNP	Lipid nanoparticles
NMD	Nonsense-mediated mRNA decay
NTC	Natural termination codons
PTC	Premature termination codons
SMA	Spinal muscular atrophy
Sup-tRNA	Nonsense suppressor tRNA
VLP	Virus-like particles
CLUSTER	Cas13 Leveraged for Undesired Sequence Toggling and Editing Reduction
